# PCA3 rs544190G>A and prostate cancer risk in an eastern Chinese population

**DOI:** 10.1590/S1677-5538.IBJU.2017.0146

**Published:** 2018

**Authors:** Dalong Cao, Chengyuan Gu, Dingwei Ye, Bo Dai, Yao Zhu

**Affiliations:** 1 Department of Urology, Fudan University Shanghai Cancer Center, Shanghai, China; 2 Department of Oncology, Shanghai Medical College, Fudan University, Shanghai, China

**Keywords:** Prostatic Neoplasms, prostate cancer antigen 3, human [Supplementary Concept], Polymorphism, Single Nucleotide

## Abstract

**Background:**

The association of prostate cancer antigen 3 (PCA3) polymorphism (SNP, rs544190G>A) with metastatic prostate cancer in European descent has been reported. Our aim of the current study was to re-validate the effect of PCA3 polymorphism on prostate cancer risk in an Eastern Chinese population and then estimate possible genetic discrepancies among population.

**Materials and Methods:**

Taqman assay was employed to determine genotype of SNP rs544190 in 1015 ethnic Han Chinese patients with prostate cancer and 1032 cancer-free controls. Simultaneously, odds ratios (OR) and 95% confidence intervals (95%CI) for risk relationship were calculated by logistic regression models.

**Results:**

The statistically significant relationship between PCA3 rs544190G>A and higher prostate cancer risk was not found. Stratification analysis revealed that there was no remarkable association of rs544190 variant AG/AA genotype with prostate cancer risk in every subgroup, except for patients with Gleason score ≤7(3+4).

**Conclusion:**

Although the results demonstrated that SNP rs544190 was not involved in prostate cancer risk in Eastern Chinese descent, unlike in European population, these might have clinical implications on prostate cancer heterogeneity around the World. To validate these findings, well-designed studies with different ethnic populations are warranted.

## INTRODUCTION

In the Western male population, prostate cancer is the most commonly diagnosed carcinoma and the second leading cause of cancer-related deaths (1). Owing to the improvement of health care system, the change of lifestyles and the extension of life expectancy, the detection rate and thus prostate cancer morbidity in China is increasing quickly (2, 3). Prostate cancer is not easier to occur in individuals of Asian ancestry than in their white and African counterparts, and Asian descent who live in diverse environment around the World still have low risk of developing prostate cancer (4–6). Based on these evidences, some genetic factors represented individual characteristics may contribute to the diverse mechanism for prostate carcinogenesis among ethnic populations. Importantly, single nucleotide polymorphisms (SNPs) have been studied and used to track cancer-causing genes (7), and thus the analysis of SNPs may assist in the development of new mechanism and agents for treating cancer.

Long non-protein-coding RNA (lncRNA) can regulate the expression of genes in close genomic proximity and target distant transcriptional activators or repressors via transcriptional interference, initiation of chromatin remodeling, pro-moter inactivation and activation of an accessory protein (8–11). Therefore, variations in lncRNAs are likely to modify functions of various biological pathways involved in prostate carcinogenesis. One of the famous lncRNA in prostate cancer is prostate cancer antigen 3 (PCA3) (12), the expression of which significantly increased in prostate tumors compared with expression in adjacent non-neoplastic prostate tissue (13). Some studies (14, 15) also revealed that the overexpression of PCA3 RNA are strongly related with malignant transformation of prostatic epithelial cells and with higher Gleason score. Therefore, further studying PCA3 polymorphism which can alter its expression and then alter its function provide clues for the clini-cal management of prostate cancer (13–16).

Recently, researchers (17) found that the PCA3 SNP rs544190 locates at the -845 locus of PCA3 and is a transition G>A in the promoter region, and then results in the increased expression of PCA3. Meanwhile, this SNP has been verified to be significantly associated with metastatic prostate cancer risk in European population (17). As is well-known that the results of associational studies may vary among populations due to inter-population genetic differences including differences in allele frequencies and linkage disequilibrium (LD) structures (18). Thus, it is more reasonable to re-evaluate the association of this SNP with prostate cancer risk in different cohort other than the one in which this association was identified.

In the current study, a case-control study was conducted to further validate the reported association of rs544190 with prostate cancer risk and other clinical characteristics in an Eastern Chinese cohort.

## MATERIALS AND METHODS

A total of 1015 ethnic Han Chinese patients with prostate cancer from Fudan University Shanghai Cancer Center (FUSCC) between 2009 and 2012 was included in the current study. Ethnic Han Chinese is one of the ethnic groups in China and constitutes approximately 92% of the population of Mainland China. All of patients were collected from Eastern China, which includes Shanghai, Zhejiang, Jiangsu and the surrounding regions. The tissue bank of FUSCC provided samples of prostate cancer patients. Additionally, 1032 cancer-free controls of ethnic Han Chinese in Eastern China were recruited from the Taizhou Longitudinal (TZL) study with the selection criteria containing no individual history of cancer (19). Controls were frequency matched to the cases on age and sex. The present study also obtained data on environmental exposure history and demographic characteristics of each participant, such as smoking status, age, sex, ethnicity, BMI. Approximately 10 mL of blood, of which 1 mL was used for genomic DNA extraction, was collected from each participant. All of study participants have signed written informed consents. The study protocol was approved by the Institutional Review Board of FUSCC (Ethical Application Ref: 050432-4-1212B). Moreover, all methods included in protocol of this study were performed in accordance with the approved guidelines.

Genomic DNA was isolated from blood sample of participant by utilizing the QIAamp DNA blood maxi kit (Qiagen, Valencia, CA). Then, the Taqman assays (Applied Biosystems, Foster City, CA) with a 7900 HT sequence detector system (Applied Biosystems) was used to determine PCA3 rs544190G>A (Forward primer: CCAATGAACTGGGATGACACAA, Reverse primer: AGGAGAGGCAAGGAAGCTAA, probes: GTTTCATATCCCCC[G/A]TCCCCAAGAGAGGA). For the quality control, each 384-plate included four negative controls (without DNA template) and two duplicated samples. Meanwhile, the assays were repeated in 5% of the samples and 100% concordance was obtained.

For the statistical analysis conducted with SAS software (version 9.1; SAS Institute, Cary, NC), X^2^ test was used to evaluate the difference in the frequency of genotype as well as demographic and other covariates between cases and controls. The good-of-fit X^2^ Test was applied to estimate the Hardy–Weinberg equilibrium of genotype distributions in the controls. By utilizing regression models, crude and adjusted odds ratios (ORs) were calculated to evaluate associations between the genotypes and prostate cancer risk. The homogeneity tests were performed to explore the difference in risk estimates among subgroups. The haplotype analysis was not performed due to the selected SNP in the same block. All tests were two-sided, and P value less than 0.05 was considered to have statistical significance.

## RESULTS

As presented in our previous paper (20), significant difference in the frequency of body mass index (BMI) between cases and controls was presented (39.15% vs 24.9%, p<0.0001), except for age and smoking status. Simultaneously, the characteristics included PSA, Gleason score, TNM stage of cases were also summarized.

In the present study, the allele and genotype frequencies of SNP rs544190 in cases and controls are listed in Table-1. Taking advantage of good-of-fit X^2^ Test, the researched genotype distributions for rs544190 among the controls were consistent with the Hardy-Weinberg equilibrium (p=0.119). However, no significant difference in the genotype distribution of PCA3 rs544190 between the cases and controls was observed (p=0.7291). Meanwhile, the rs544190 A allele was less frequent in controls than in cases with no significantly statistical difference (p=0.7012). In multivariate logistic regression analysis, rs544190 AG, AA and AG/AA genotypes were not correlated with significantly increased risk of prostate cancer (adjusted OR=0.988, 95%CI=0.821-1.190, p=0.8993; adjusted OR=1.123, 95%CI=0.806-1.564, p=0.494; and adjusted OR=1.011, 95% CI=0.849-1.205, p=0.9019, respectively) when the genotype GG was used as reference and age, smoking and BMI were adjusted. Compared with the genotypes (GG/AG), there was also no significant relationship between rs544190 AA genotype and the increased prostate cancer risk in the recessive model (adjusted OR = 1.128, 95% CI=0.816-1.559, p=0.4663).

**Table 1:** Logistic regression analysis of associations between PCA3 variant genotype and prostate cancer risk.

Variables	Genotypes	Cases no. (%)	Controls no. (%)	P^a^	Crude OR (95% CI)	Adjusted OR (95% CI)^b^	P^b^
**rs544190**							
	GG	573 (56.45)	584 (56.59)	0.7291	1.00	1.00	
	AG	358 (35.27)	372 (36.05)		0.981(0.815-1.181)	0.988(0.821-1.190)	0.8993
	AA	84 (8.28)	76 (7.36)		1.126(0.809-1.568)	1.123(0.806-1.564)	0.494
	AG+AA	442 (43.55)	448 (43.41)	0.9505^d^	1.006(0.844-1.198)	1.011(0.849-1.205)	0.9019
**Additive model**				0.7291^c^	1.026(0.896-1.176)	1.028(0.897-1.178)	0.6871
	GG+AG	931 (91.72)	956 (92.64)		1.00	1.00	
	AA	84 (8.28)	76 (7.36)	0.4424^e^	1.135(0.821-1.568)	1.128(0.816-1.559)	0.4663
**A allele frequency**		0.2591	0.2539	0.7012			

Abbreviation: PCA3, Prostate Cancer Antigen 3; OR, odds ratio; 95% CI, 95% confidence interval; rs, reference single nucleotide polymorphism.

a Chi-square tests were used to calculate differences for the frequency distribution of genotypes, combined genotypes, or alleles between cases and controls;

b Adjusted for age, smoking, and BMI status in logistic regress models;

c For additive genetic models;

d For dominant genetic models;

e For recessive genetic models.

The results were in bold, if *P*<0.05 or 95%CI excluded 1.

Next, the correlation of the SNP rs544190 with prostate cancer risk was furtherly assessed by stratified analysis using age, BMI, smoking, Gleason score, TNM stage and aggressive status (Table-2). Nevertheless, the notable relationship between prostate cancer risk and rs544190 variant AG/AA genotypes was also not appeared in these strata. Meanwhile, there was non-significant difference in risk estimates among these strata in further heterogeneity test, except for Gleason score ≤7(3+4). The results mentioned above indicated that there might exist potential correlation of PCA3 rs544190 with Gleason score.

**Table 2:** Stratification analysis for associations between PCA3 variant and prostate cancer risk by dominant genetic model in all subjects of Eastern chinese man.

variables		rs544190 (cases/controls)		Adjusted OR(95%CI)^a^	*P* a	*P* ^hom^
		AA+AG	GG			
**Age, year**						
	≤69 (mean)	232/242	282/287	0.976(0.746-1.247)	0.8454	0.719
	>69 (mean)	210/206	291/297	1.045(0.813-1.344)	0.7315	
**BMI, kg/m^2^**						
	≤25	323/271	439/357	0.970(0.783-1.201)	0.7781	0.407
	>25	119/177	134/227	1.143(0.833-1.567)	0.4078	
**Smoking status**						
	Never	183/175	223/231	1.100(0.833-1.453)	0.5019	0.496
	Ever	259/273	350/353	0.957(0.763-1.199)	0.7010	
**Gleason score**						
	≤7(3+4)	155/448	162/584	1.254(0.974-1.615)	0.0793	0.046
	≥7(4+3)	247/448	359/584	0.898(0.732-1.101)	0.3006	
**Stage of disease**						
	I+II	199/448	240/584	1.085(0.866-1.359)	0.4774	0.336
	III+IV	207/448	291/584	0.928(0.747-1.152)	0.4979	
**Aggressive** b						
	low	109/448	117/584	1.205(0.902-1.609)	0.2066	0.165
	high	333/448	456/584	0.955(0.792-1.152)	0.6307	

**PCA3** = Prostate Cancer Antigen 3; **rs** = reference single nucleotide polymorphism; **OR** = odds ratio; 95% **CI** = 95% confidence interval; **P hom** = Homogeneity test; **BMI** = body mass index.

a Obtained in logistic dominant models with adjustment for age, smoking status and BMI;

b Lowly aggressive status refers to Gleason score <7 or PSA ≤10ng/mL or cT stage <cT2b; Highly aggressive status refers to Gleason score ≥7 or PSA >10ng/mL or cT stage ≥cT2b.

The results were in bold, if *P*<0.05.

## DISCUSSION

The association of prostate cancer risk with PCA3 polymorphism -845G>A was mainly determined in the present study, and also was firstly evaluated in an Eastern Chinese population.

Increasing evidences (21–23) have shown that lncRNAs are involved in transcription, splicing, translation, protein localization, cellular structure integrity, imprinting, cell cycle and apoptosis, stem cell pluripotency and reprogramming and heat shock response. Furthermore, researchers have found that lncRNAs play a very vital role in pathological conditions such as cancer and cardiovascular disease and consequently provide novel biomarkers and pharmaceutical targets (24, 25). To the best of our knowledge, polymorphisms which are responsible for change in gene expression or function can be characterized as functional genetic variants (26). These evidences mentioned above have indicated that variation in lncRNA regions may contribute to the etiology of disease.

In the current study, the rs544190 AA genotype of PCA3, as one of lncRNAs, was not detected to be correlated with increased prostate cancer risk. Meanwhile, there was also non-significant association of genotype AG and AG/AA with the higher risk of prostate cancer. However, a previous study showed that A allele carriers possess an increased risk for developing metastatic prostate cancer (17). Differences in research participants, sample size and ancestral background might lead to inconsistent results among various studies. To further research the relationship be-tween previously reported risk loci and prostate cancer risk instead of metastatic prostate cancer risk, the present study with the larger number of subjects was timely designed and conducted.

By stratified analysis using age, BMI, smoking, Gleason score, TNM stage and aggressive status, the statistically significant association of PCA3 rs544190 with increased prostate cancer risk in every subgroup was not found. Other studies (13, 17) have also indicated no significant relationship between allele and elevated Gleason score in prostate cancer. Such findings mentio-ned above might be interpreted by the fact that there was still not enough number of participants to provide statistical power to detect any relationship in stratified analysis. Additionally, cancer is a complex and multifactorial disease so that a single genetic variant is insufficient to predict the overall risk. Therefore, larger number of participants and more SNPs in lncRNAs or in other related gene related with the etiology of prostate cancer should be considered in the future studies.

Here, limitations in the current study need to be addressed. Firstly, bias from selection of the non-representative population would not be absolutely excluded because it was a hospital-based case-control study with patients from FUSCC and controls from TZL study (19). By means of frequency-matching cases and controls on age, areas of residence and further adjustment for possible confounding factors in final analysis, potential confounding bias might be furthest minimized. Secondly, owing to the nature of retrospective study design, reliable and sufficient information about exposure data were not available. Finally, genetic variations discovered through association studies are rarely the actual causal variant-rather, they may/mayn't be associated with disease risk for linkage disequilibrium which sometimes extends over relatively large distances in the human genome (27). Thus, an integrated and systematic approach needs to explore the mechanisms that increase the risk for cancer. In the future, these limitations would be conquered via larger, well designed and prospective population-based studies.

## CONCLUSIONS

Although the current study has found that the polymorphism PCA3 -845 G>A was not correlated with progression to prostate cancer in an Eastern Chinese population, this might have clinical implications on prostate cancer heterogeneity around the World. Larger and more in-depth molecular studies for exploring the role of rs544190G>A in prostate cancer are warranted before it may be contributed to clinical decision-making.
